# Detection of Cell-Free Mitochondrial DNA in Cerebrospinal Fluid of Creutzfeldt-Jakob Patients

**DOI:** 10.3389/fneur.2019.00645

**Published:** 2019-06-21

**Authors:** Jie Li, Yuhan Duan, Deming Zhao, Syed Zahid Ali Shah, Wei Wu, Xixi Zhang, Mengyu Lai, Zhiling Guan, Dongming Yang, Xiaoqian Wu, Hongli Gao, Huafen Zhao, Qi Shi, Lifeng Yang

**Affiliations:** ^1^Key Laboratory of Animal Epidemiology and Zoonosis, Ministry of Agriculture, National Animal Transmissible Spongiform Encephalopathy Laboratory, College of Veterinary Medicine, China Agricultural University, Beijing, China; ^2^Department of Pathology, Faculty of Veterinary Sciences, Cholistan University of Veterinary and Animal Sciences, Bahawalpur, Pakistan; ^3^State Key Laboratory for Infectious Disease Prevention and Control, Chinese Center for Disease Control and Prevention, National Institute for Viral Disease Control and Prevention, Beijing, China

**Keywords:** mtDNA, Creutzfeldt-Jakob disease, cerebrospinal fluid, diagnosis, prion disease

## Abstract

**Background:** The current diagnosis method for Creutzfeldt-Jakob disease (CJD) is post-mortem examination, so early detection of CJD has been historically problematic. Auxiliary detection of CJD based on changes in levels of components of the cerebrospinal fluid (CSF) has become a focus of research. In other neurodegenerative diseases such as Alzheimer's disease (AD), cell-free mitochondrial DNA (mtDNA) in the CSF of patients may serve as a biomarker that could facilitate early diagnosis and studies of the mechanisms underlying the disease.

**Methods:** In this study, the cell-free mitochondrial DNA in the CSF of patients with sCJD and control patients was compared by digital droplet PCR.

**Results:** The cell-free mitochondrial DNA copy number in the CSF of sCJD patients was significantly increased in comparison with that of the control group, and this difference was pathologically related to CJD.

**Conclusion:** Therefore, we speculate that changes in cerebrospinal fluid mitochondrial DNA copy number play an important role in the study of CJD mechanism and diagnosis.

## Introduction

Creutzfeldt-Jakob disease (CJD) is a neurodegenerative disease associated with an abnormal accumulation of proteinaceous infectious particles (prions) in neurons (1). CJD is the most frequent human transmissible spongiform encephalopathies (2–4). CJD can be confirmed by subjecting brain tissue from CJD patients to western blotting after digestion with proteases that is a demonstration of the protease resistance of the prion protein accumulated, or to use immunohistochemistry, abnormal PrP can be identified by its abundance, location and morphology (5). A diagnosis of pre-mortem CJD can be made based on autosomal dominant pathogenic mutations in the human prion protein gene (*PRNP)* (6) and using real-time quaking induced conversion (RT-QuIC) which detected the accumulation of misfolded PrP in the brain, CSF, or nasal brushings (7), combined with clinical manifestations, medical history, epidemiological reports, EEG, and MRI (8, 9). In recent years, non-invasive and less invasive detection methods have been used to diagnose CJD, and this idea can also be applied to the study of the mechanisms underlying CJD. CSF is in direct contact with the brain parenchyma and can indirectly reflect certain pathological indications of the central nervous system (10). Changes in the abundance of many proteins (14-3-3 protein, tau et al.) in CSF can be used in the diagnosis of CJD (11–14). RT-QuIC has proved to be a very valuable diagnostic CSF test for sCJD (7). The cell-free mitochondrial DNA content in CSF was found to be changed in a specific manner in patients with AD and PD, and such changes were distinct to particular neurodegenerative diseases (15–19). These findings suggest that particular changes in mitochondrial DNA content may be related to the mechanisms underlying specific diseases. In comparison with other neurodegenerative diseases, there have been few studies of changes in mitochondrial DNA in the CSF of patients with CJD (16).

In this study, we examined the mitochondrial DNA concentration in the CSF of sCJD patients, with the goal of determining the mechanisms underlying the development and progression of CJD and identifying if changes in cerebrospinal fluid mitochondrial DNA copy number could be suitable for diagnosing the disease.

## Materials and Methods

### Subjects

CSF samples from 20 age-and sex ratio-matched sCJD and 13 age-and sex ratio-matched non-CJD controls were presented to the Chinese center for disease control and prevention. Both the control group and the CJD patient in the sample were Chinese. The average age of the sCJD and control patients was ~55 years, and the ratio of males to females was nearly 1:1. The clinical symptoms of the subjects were: rapid progressive dementia, myoclonus, akinetic mutism, disturbance of consciousness, mental symptoms, etc. Detailed samples background information was shown in Table 1. The diagnosis of sCJD refers to the CJD diagnostic criteria standardized by the Chinese Center for Disease Control and Prevention. The CJD diagnostic criteria unified by the centers for disease control are as follows: ① Suspected cases are based on the exclusion of other diseases, and meet the following clinical manifestations 2 or more: rapid progressive dementia, passive silence, myoclonus, visual/cerebellar dysfunction, cone system/extrapyramidal dysfunction. ② The clinical cases are basically consistent with the suspected diagnosis cases, and meet any of the following items: EEG shows periodic three-phase waves during the course of the disease; laboratory tests for positive detection of 14-3-3 protein in cerebrospinal fluid (CSF); Early nuclear magnetic resonance imaging revealed abnormally high signals in the putamen/tail caudate nucleus, and diffusion-weighted images (DWI) showed a “ribbon” sign of symmetry/asymmetry cortex (or cortex). ③ The confirmed cases should meet one of the following items: spongiform lesions found in brain histopathology; immunohistochemical detection of brain tissue, presence of protease-resistant PrP^sc^ deposition; protein immunoblotting to detect brain tissue, protease-resistant PrP^sc^.

**Table 1:** Cerebrospinal fluid sample background information.

***N***	**Population distribution**		**Clinical test**								**Clinical diagnosis**
	**Gender**	**Age**	**1**	**2**	**3**	**4**	**5**	**6**	**Clinical performance**	**14-3-3 protein**	
1	Male	56	+	–	+	+	–	–	1,3,4	+	sCJD
2	Female	67	+	–	–	–	–	–	1	+	sCJD
3	Male	40	+	+	–	–	+	+	1,2,5,6	+	sCJD
4	Female	58	–	–	–	+	–	+	4,6	+	sCJD
5	Female	79	+	+	+	+	+	+	1,2,3,4,5,6	–	sCJD
6	Female	57	+	–	–	–	–	–	1	–	sCJD
7	male	43	+	–	+	+	–	+	1,3,4,6	+	sCJD
8	Male	55	+	+	+	+	+	–	1,2,3,4,5	+	sCJD
9	Female	65	+	–	+	+	–	–	1,3,4	+	sCJD
10	Male	52	+	+	–	–	+	–	1,2,5	+	sCJD
11	Female	62	+	+	+	+	+	+	1,2,3,4,5	+	sCJD
12	Male	71	+	+	+	+	–	–	1,2,3,4	+	sCJD
13	Female	57	+	+	+	–	–	–	1,2,3	+	sCJD
14	Male	51	+	–	+	+	–	–	1,3,4	+	sCJD
15	Male	64	+	+	–	+	–	+	1,2,4,6	+	sCJD
16	Female	63	–	+	–	+	+	–	2,4,5	+	sCJD
17	Male	72	+	+	–	+	+	–	1,2,4,5	+	sCJD
18	Female	71	+	+	–	+	+	–	1,2,4,5	+	sCJD
19	Male	66	+	+	+	–	–	+	1,2,3,6	+	sCJD
20	Female	54	+	+	–	+	+	+	1,2,4,5,6	+	sCJD
21	Male	62	+	–	–	–	+	–	1,5	–	non-CJD
22	male	44	+	–	–	+	–	–	1,4	–	non-CJD
23	Female	50	–	–	+	+	–	+	3,4,6	–	non-CJD
24	Male	47	–	–	–	–	–	–	/	–	non-CJD
25	Male	65	+	–	–	–	–	–	1	–	non-CJD
26	Female	76	+	–	–	+	–	–	1,4	–	non-CJD
27	Female	62	+	+	–	–	–	–	1,2	–	non-CJD
28	Female	56	+	–	+	–	–	–	1,3	–	non–CJD
29	Male	45	–	–	–	–	–	+	6	–	non-CJD
30	Male	73	+	+	–	–	–	–	1,2	–	non-CJD
31	Female	52	–	–	–	–	–	+	6	–	non-CJD
32	Male	70	+	–	–	+	–	–	1,4	–	non-CJD
33	Female	55	–	–	–	+	–	–	4	–	non-CJD

“*1” represent “Rapid progressive dementia,” “2” represent “Myoclonus,” “3” represent “Visual or cerebellar symptoms,” “4” represent “Pyramids/Extrapyramidal dysfunction,” “5” represent “akinetic mutism,” “6” represent “other symptoms.” “/” represent “inexistence,” “Other symptoms” include paroxysmal convulsions, disturbance of consciousness, convulsions, slow progressive dementia, psychiatric symptoms, subacute progressive dementia, dual vision, cortical blindness, numbness of the right limb, dizziness, nausea, vomiting, memory loss, cerebellar symptoms. “+” represent “exist,” “–” represent “inexistence.”*

### Detection of CSF Mitochondrial DNA Copy Number in sCJD Patients

#### Digital Droplet Polymerase Chain Reaction (ddPCR)

The CSF mtDNA copy number of sCJD patients and non-CJD samples was directly determined by ddPCR. In this experiment, an amplification product containing 85 base pairs (mtDNA-85) was the only product generated. Three replicates were performed in 96-well plates. The sequences of the amplified mtDNA primer and probe were shown in Supplementary Table 1-1 and Date Supplementary Table 1-2. The amplification reaction system and amplification reaction procedure were shown in Date Supplementary Tables 1-3, 1-4. After the amplification reaction was complete, the QX200 Droplet Reader was used to read the signal, after which the data were analyzed.

#### Statistical Analysis

Statistical analysis was performed using Graphpad Prism 7 software to compare the mitochondrial DNA copy number in CSF samples from the control group and sCJD patients. At the same time, the factors of gender, age, and symptoms were analyzed to assess their relationship with the CSF mitochondrial DNA copy number. The results are expressed as the mean mtDNA concentration in CSF (mtDNA copy/20 μL ± SEM). The statistical significance was set at *p* < 0.05.

## Results

### Mitochondrial DNA Copy Number of the CSF of sCJD Patients (Calculated per 20 μL Copy Number)

**Figure 1:**
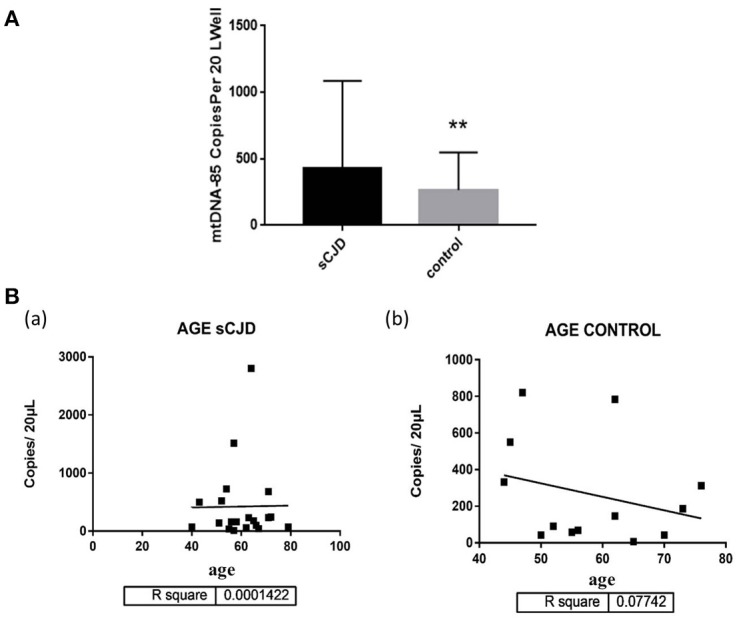
**(A)** The mitochondrial DNA copy number of the CSF of the control group (*n* = 13) and patients with sCJD (*n* = 21). Subjects were divided into the following groups: possible sCJD patients (black bar) and non-CJD subjects (gray bar). The results are expressed as mean ± SEM. The mtDNA copy number of the CSF is expressed as mtDNA copies/20 μL. ^*^P < 0.05; ^**^P < 0.01, represents the sCJD group are significantly different from the control group, by unpaired *t*-test. **(B)** mitochondrial DNA copy numbers of the CSF of sCJD patients/non-patients of different ages. The correlation between age and the cell-free mitochondrial DNA copy number of the CSF was assessed for the experimental and control groups (a,b). There was no correlation between age and disease, By liner regression graph and pearson correlation analysis.

The amplified mitochondrial DNA copy number of CSF in the experimental group and the control group results are shown in Supplementary Table 2. The mitochondrial DNA copy number of the CSF of sCJD patients (404.9 ± 142.2 copies/20 μL) was significantly increased in comparison with that of the control group (264.3 ± 78.62 copies/20 μL) (Figure 1A).

### Correlation Between Changes in Mitochondrial DNA Copy Number and age in Cerebrospinal Fluid in Patients With Creutzfeldt-Jakob

We further evaluated the association between changes in mitochondrial DNA copy number and age in cerebrospinal fluid in CJD patients. The mitochondrial DNA copy number of the CSF was not associated with age in the sCJD or control group (Figure 1B).

### Correlation Between Changes in Mitochondrial DNA Copy Number and Gender in Cerebrospinal Fluid in Patients With Creutzfeldt-Jakob

**Figure 2:**
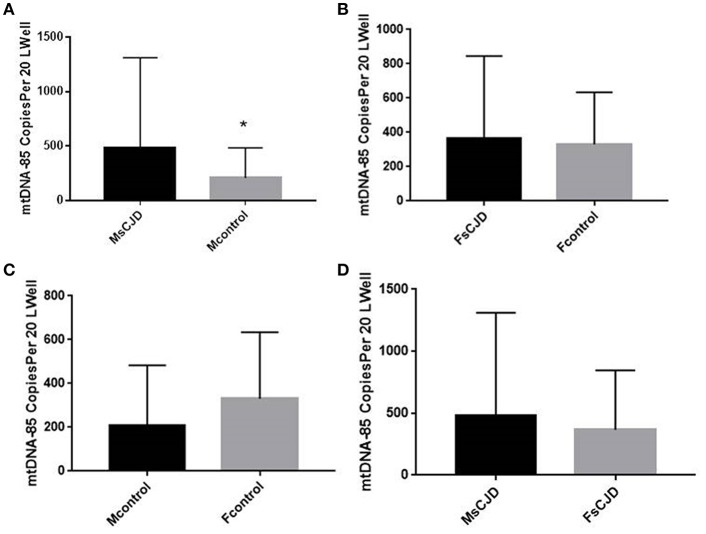
Copies of mtDNA in CSF and correlation with gender. **(A)** The mitochondrial DNA copy number of the male sCJD patients was significantly higher than that of the control group. **(B)** The mitochondrial DNA copy number of the female sCJD patients (*n* = 11) was higher than that of the control group (*n* = 10), but this difference was not significant. **(C)** In the control group, the cell-free mitochondrial DNA copy number of female patients (*n* = 6) was higher than that of male patients (*n* = 7), but this difference was not significant. **(D)** Among sCJD patients, the mitochondrial DNA copy number of male patients was higher than that of female patients, but this difference was not significant. The results are expressed as mean ± SEM. The mtDNA copy number of the CSF is expressed as mtDNA copies/20 μL. ^*^P < 0.05; ^**^P < 0.01, represents the MsCJD group are significantly different from the control group, by unpaired *t*-test.

We also evaluated the association between changes in cerebrospinal fluid mitochondrial DNA copy number and gender in Creutzfeldt-Jakob patients. The mitochondrial DNA copy number of male sCJD patients (480.2 ± 263.1 copies/20 μL) was significantly higher than that of the control group (208.1 ± 103.9 copies/20 μL), and this difference was significant. The mitochondrial DNA copy number of female sCJD patients (367.3 ± 151.1 copies/20 μL) was higher than that of the control group (330 ± 124.2 copies/20 μL), but this difference was not significant. In the control group, the cell-free mitochondrial DNA copy number of the CSF of female patients (330 ± 124.2 copies/20 μL) was higher than that of male patients (208.1 ± 103.9 copies/20 μL), but this difference was not significant. Among the sCJD patients, the mitochondrial DNA copy number of the male patients (480.2 ± 263.1 copies/20 μL) was higher than that of the female patients (367.3 ± 151.1 copies/20 μL), but this difference was not significant (Figure 2).

### Correlation Between Changes in Mitochondrial DNA Copy Number and Clinical Symptoms in Cerebrospinal Fluid in Patients With Creutzfeldt-Jakob

**Figure 3:**
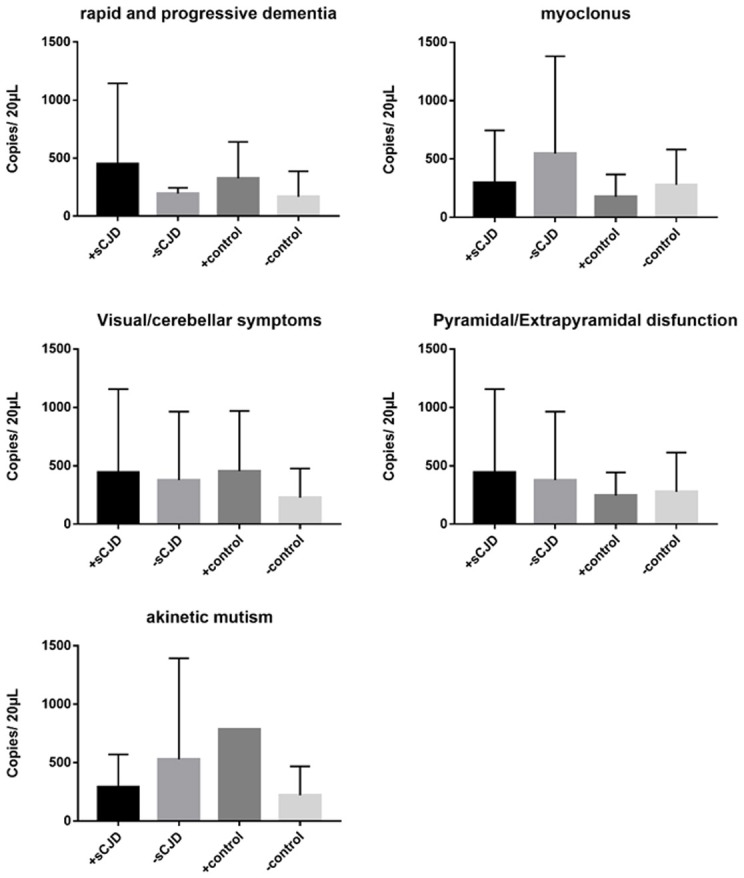
The correlation between clinical symptoms and the cell-free mitochondrial DNA copy number of the CSF was analyzed for the experimental and control groups. The CSF mitochondrial DNA copy number was not found to be related to CJD symptoms. The results are expressed as mean ± SEM. The mtDNA copy number of the CSF is expressed as mtDNA copies/20 μL. ^*^P < 0.05; ^**^P < 0.01, represents the sCJD group are significantly different from the control group, by unpaired *t*-test.

We next evaluated the association between changes in cerebrospinal fluid mitochondrial DNA copy number and clinical symptoms in Creutzfeldt-Jakob patients. Clinical symptoms include rapid progressive dementia, myoclonus, akinetic mutism, visual/cerebellar symptoms, pyramidal/extrapyramidal dysfunction, mental symptoms. The mitochondrial DNA copy numbers of the CSF of the experimental and control groups were not significantly associated with differences in clinical symptoms (Figure 3).

### Correlation Between Changes in Mitochondrial DNA Copy Number and 14-3-3 Protein in Cerebrospinal Fluid in Patients With Creutzfeldt-Jakob

Finally, we examined the association between the copy number of mitochondrial DNA and 14-3-3 protein in CSF. The mitochondrial DNA copy number of the 14-3-3 protein positive group (466.3 ± 161.9 copies/20 μL) was significantly higher than that of the 14-3-3 protein negative group (234.5 ± 70.81 copies/20 μL). Statistical analysis results were shown in Supplementary Figure 1.

## Discussion

Here, we investigated changes in the concentration of mtDNA in CSF from sCJD patients. In comparison with the control group with general neurological symptoms, the increase in the mitochondrial DNA copy number of the CSF of patients with sCJD was significant. Therefore, we speculate that changes in cerebrospinal fluid mitochondrial DNA copy number play an important role in the study of CJD mechanism and diagnosis. Our data support previous work where, other than in AD, all the other non-AD type dementia, including sCJD have an increased of relative mtDNA copy numbers (16). They also found that the 14-3-3 protein was not associated with significant differences in mitochondrial DNA copy number in CSF. However, our experimental results showed that the mitochondrial DNA copy number was significantly increased in the 14-3-3 protein-positive group compared to the 14-3-3 protein-negative group, and in 20 cases of CJD cerebrospinal fluid samples, 18 samples of 14-3-3 protein were positive. In general, this result indicated that the change in mitochondrial DNA copy number has a certain correlation with 14-3-3 protein. We hypothesized that changes in mitochondrial DNA copy number are important for the diagnosis of CJD.

This study did not find a relationship between cell-free mitochondrial DNA content in CSF and the age/clinical neurological symptoms of CJD or non-CJD patients, which is similar to PD patients (18). However, Wei et al.'s research indicated that changes in mitochondrial DNA copy number in brain samples from Creutzfeldt-Jakob patients were strongly positively correlated with age (20). We speculate that because our sample size is small and the age span is small, our sample age is concentrated around 55 years old. The mitochondrial DNA copy number changes in this period of time are relatively small. Although they were different, they were not significant between each other, and the results obtained were one-sided.

The cell-free mitochondrial DNA copy number of the CSF of male patients was significantly increased compared with that of the control group, and it was significantly correlated with sCJD. However, there was no correlation between mitochondrial DNA copy number and sCJD in female patients. Therefore, changes in cerebrospinal fluid mitochondrial DNA copy number in male CJD patients play a better role than in female patients in studying CJD mechanisms and diagnosis. This phenomenon has also been observed in PD, AD, and Huntington's disease patients, in which mitochondrial DNA in men is more closely associated with the disease (18, 21–23).

We speculate that the extent of the increase in mitochondrial DNA in CSF directly reflects neuronal damage. When mitochondria are dysfunctional, an insufficient energy supply leads to neuronal degeneration, and an increased mitochondrial DNA copy number in CSF may be a response to neuronal mitochondrial dysfunction to maintain neuronal energy requirements. This hypothesis is consistent with the conclusion of Wei et al. (20) they observed a strongly positive correlation between age and mtDNA copy number in CJD, this could reflect compensatory mitochondrial biogenesis in older subjects. There are no effective prevention or treatment methods for most neurodegenerative diseases. Changes in the number of mitochondrial DNA copies are associated with several neurodegenerative diseases, including sCJD, PD, and AD, so we hypothesize that drugs that regulate the mtDNA copy number in neurons may have therapeutic effects in patients with certain neurodegenerative diseases.

## Conclusions

The mtDNA-85 copy number in cerebrospinal fluid of patients with sCJD was significantly different from that of the control group, we speculate that changes in cerebrospinal fluid mitochondrial DNA copy number play an important role in the study of CJD mechanism and diagnosis. Due to the small number of samples we collected, so our results are indeed limited, we need a larger sample to further verify this result.

## Data Availability

We declared that materials described in the manuscript, including all relevant raw data, will be freely available to any scientist wishing to use them for non-commercial purposes, without breaching participant confidentiality.

## Ethics Statement

The study was ethically approved by the Institutional Ethics committee of the Chinese Center for Disease Control and Prevention. Written informed consent was obtained from each participant.

## Author Contributions

JL, YD, SS, and LY conceived the study. WW, XZ, ML, and QS collected the CSF samples and clinical data. JL, YD, ZG, HG, HZ, XW, and DY performed the experiments and data analyses. LY and DZ provided intellectual inputs. JL and YD wrote the manuscript. All authors read and approved the manuscript.

### Conflict of Interest Statement

The authors declare that the research was conducted in the absence of any commercial or financial relationships that could be construed as a potential conflict of interest.

## Abbreviations

AD: Alzheimer's disease
CJD: Creutzfeldt-Jakob disease
CSF: cerebrospinal fluid
ddPCR: digital droplet polymerase chain reaction
mtDNA: mitochondrial DNA
PD: Parkinson's Disease
sCJD: Sporadic Creutzfeldt-Jakob disease
TSEs: Transmissible spongiform encephalopathies.
