# Supplementary material for: Detection of Cell-Free Mitochondrial DNA in Cerebrospinal Fluid of Creutzfeldt-Jakob Patients
Source: Front Neurol. 2019 Jun 21;10:645. doi: 10.3389/fneur.2019.00645 (PMC6598448; doi:10.3389/fneur.2019.00645)
Supplement: Supplementary file 1 [file Table_1.DOCX]

**Supplementary table 1 :** The sequences of the amplified mtDNA primer and probe

supplementary table 1-1 Digital Droplet PCR Primer Synthesis Sequence and Information

| **Primer sequence (5′–3′)** | **Primer name** | **5′ modification** | **3′ modification** | **Purification method** |
| --- | --- | --- | --- | --- |
| **CTCACTCCTTGGCGCCTGCC** | mitochondrial DNA-85 upstream primers |  |  | iPAGE |
| **GGCGGTTGAGGCGTCTGGTG** | Mitochondrial DNA-85 downstream primers |  |  | iPAGE |
| **CCTCCAAATCACCACAGGACTA**  **TTCCTAGCCATGCA** | Mitochondrial DNA-85 fluorescent probe | FAM | BQ1 | HPL |

supplementary table 1-2 Digital Droplet PCR Sample Amplification Information

| **Detection of genetic information** | **Name of amplification primer** | **Probe Type** |
| --- | --- | --- |
| Mitochondrial DNA - 85 | Mitochondrial DNA – 85-F | FAM |
|  | Mitochondrial DNA - 85 -R |  |

supplementary table 1-3 Amplification Reaction System

| **PCR reaction system** | **Loading amount (L)** |
| --- | --- |
| **2×PCR Mix** | **10.0** |
| **Primer/Probe Mix** | **1.0** |
| **H2O** | **6** |
| **Template (1)** | **3** |

supplementary table 1-4 Amplification reaction procedure

| **Stage** | **Temperature** | **Time** | **Cycling number** |
| --- | --- | --- | --- |
| **Holding Stage** | **95°C** | **10 min** | **1 cycle** |
| **Cycling Stage** | **94°C** | **30 s** | **40 cycles** |
|  | **60°C** | **1 min** |  |
| **Holding Stage** | **98°C** | **10 min** | **1 cycle** |
| **Holding Stage** | **4°C** | **∞** |  |
