# Supplementary material for: Detection of Cell-Free Mitochondrial DNA in Cerebrospinal Fluid of Creutzfeldt-Jakob Patients
Source: Front Neurol. 2019 Jun 21;10:645. doi: 10.3389/fneur.2019.00645 (PMC6598448; doi:10.3389/fneur.2019.00645)
Supplement: Supplementary file 2 [file Table_2.DOCX]

**Supplementary table 2:** The amplified mitochondrial DNA copy number of CSF in the experimental group and the control group results.

Supplementary table 2 Digital droplet PCR results (calculated per 20 μL copy number)

| **Sample** | **Target** | **Copies Per 20 μL Well** | **Positives** | **Negatives** | **Accepted Droplets** | **Loading amount (μL)** |
| --- | --- | --- | --- | --- | --- | --- |
| **S-1** | **mitochondrial DNA-85** | **160** | **122** | **17923** | **18045** | **3** |
| **S-2** | **mitochondrial DNA-85** | **44** | **37** | **19683** | **19720** | **3** |
| **S-3** | **mitochondrial DNA-85** | **70** | **35** | **11814** | **11849** | **1** |
| **S-4** | **mitochondrial DNA-85** | **160** | **93** | **13559** | **13652** | **2** |
| **S-5** | **mitochondrial DNA-85** | **70** | **47** | **15967** | **16014** | **0.5** |
| **S-6** | **mitochondrial DNA-85** | **11.2** | **8** | **16705** | **16713** | **2** |
| **S-7** | **mitochondrial DNA-85** | **498** | **367** | **17172** | **17539** | **2** |
| **S-8** | **mitochondrial DNA-85** | **36** | **23** | **15417** | **15440** | **2** |
| **S-9** | **mitochondrial DNA-85** | **178** | **82** | **10857** | **10939** | **2** |
| **S-10** | **mitochondrial DNA-85** | **522** | **409** | **18234** | **18643** | **2** |
| **S-11** | **mitochondrial DNA-85** | **58** | **46** | **18354** | **18400** | **2** |
| **S-12** | **mitochondrial DNA-85** | **234** | **116** | **11612** | **11728** | **2** |
| **S-13** | **mitochondrial DNA-85** | **1514** | **904** | **13598** | **14502** | **2** |
| **S-14** | **mitochondrial DNA-85** | **140** | **93** | **15582** | **15675** | **2** |
| **S-15** | **mitochondrial DNA-85** | **2800** | **1410** | **11195** | **12605** | **2** |
| **S-16** | **mitochondrial DNA-85** | **230** | **126** | **12877** | **13003** | **1** |
| **S-17** | **mitochondrial DNA-85** | **242** | **121** | **11721** | **11842** | **2** |
| **S-18** | **mitochondrial DNA-85** | **682** | **434** | **14758** | **15192** | **2** |
| **S-19** | **mitochondrial DNA-85** | **100** | **57** | **13275** | **13332** | **1** |
| **S-20** | **mitochondrial DNA-85** | **726** | **385** | **12285** | **12670** | **2** |
| **Contrast-21** | **mitochondrial DNA-85** | **784** | **555** | **16391** | **16946** | **3** |
| **Contrast-22** | **mitochondrial DNA-85** | **186** | **153** | **19286** | **19439** | **3** |
| **Contrast-23** | **mitochondrial DNA-85** | **90** | **77** | **20168** | **20245** | **3** |
| **Contrast-24** | **mitochondrial DNA-85** | **42** | **31** | **17649** | **17680** | **2** |
| **Contrast-25** | **mitochondrial DNA-85** | **58** | **42** | **17152** | **17194** | **2** |
| **Contrast-26** | **mitochondrial DNA-85** | **332** | **235** | **16549** | **16784** | **2** |
| **Contrast-27** | **mitochondrial DNA-85** | **42** | **32** | **17579** | **17611** | **2** |
| **Contrast-28** | **mitochondrial DNA-85** | **820** | **608** | **17136** | **17744** | **2** |
| **Contrast-29** | **mitochondrial DNA-85** | **6.4** | **5** | **18103** | **18108** | **2** |
| **Contrast-30** | **mitochondrial DNA-85** | **312** | **244** | **18290** | **18534** | **2** |
| **Contrast-31** | **mitochondrial DNA-85** | **146** | **105** | **16786** | **16891** | **2** |
| **Contrast-32** | **mitochondrial DNA-85** | **68** | **51** | **17699** | **17750** | **2** |
| **Contrast-33** | **mitochondrial DNA-85** | **550** | **423** | **17909** | **18332** | **2** |
