# Supplementary material for: Detection of Cell-Free Mitochondrial DNA in Cerebrospinal Fluid of Creutzfeldt-Jakob Patients
Source: Front Neurol. 2019 Jun 21;10:645. doi: 10.3389/fneur.2019.00645 (PMC6598448; doi:10.3389/fneur.2019.00645)
Supplement: Supplementary file 3 [file Data_Sheet_1.docx]

**Supplementary figure 1:** Statistical analysis of the correlation between mitochondrial DNA and 14-3-3 protein in cerebrospinal fluid.

Supplementary figure 1

Supplementary figure1. The correlation between mitochondrial DNA and 14-3-3 protein in cerebrospinal fluid. The mitochondrial DNA copy number of the 14-3-3 protein positive group(n=18) was significantly higher than that of the 14-3-3 protein negative group(n=15). The results were expressed as mean ± SEM. The mtDNA copy number of the CSF is expressed as mtDNA copies/20 μL. *P <0.05; **P <0.01, significantly different from the control group, by unpaired t test.
